# Stromal CD38 regulates outgrowth of primary melanoma and generation of spontaneous metastasis

**DOI:** 10.18632/oncotarget.25737

**Published:** 2018-08-07

**Authors:** Bar Ben Baruch, Eran Blacher, Einav Mantsur, Hila Schwartz, Hananya Vaknine, Neta Erez, Reuven Stein

**Affiliations:** ^1^ Department of Neurobiology, George S. Wise Faculty of Life Sciences, Tel Aviv University, Tel Aviv, Israel; ^2^ Department of Pathology, Sackler School of Medicine, Tel Aviv University, Tel Aviv, Israel; ^3^ Department of Pathology, Wolfson Medical Center and Sackler Faculty of Medicine, Tel Aviv University, Holon, Israel

**Keywords:** CD38, melanoma, tumor microenvironment, primary tumor, metastasis

## Abstract

The outgrowth of primary melanoma, the deadliest skin cancer, and generation of metastasis is supported by the tumor microenvironment (TME) which includes non-cancerous cells. Since the TME plays an important role in melanoma pathogenesis, its targeting is a promising therapeutic approach. Thus, it is important to identify proteins in the melanoma TME that may serve as therapeutic targets. Here we show that the nicotinamide adenine dinucleotide glycohydrolase CD38 is a suitable target for this purpose. Loss of CD38 in the TME as well as inhibition of its enzymatic activity restrained outgrowth of primary melanoma generated by two transplantable models of melanoma, B16F10 and Ret-mCherry-sorted (RMS) melanoma cells. Pathological analysis indicated that loss of CD38 increased cell death and reduced the amount of cancer-associated fibroblasts (CAFs) and blood vessels. Importantly, in addition to inhibiting outgrowth of primary melanoma tumors, loss of CD38 also inhibited spontaneous occurrence of RMS pulmonary and brain metastasis. The underlying mechanism may involve, at least in the brain, inhibition of metastasis expansion, since loss of CD38 inhibited the outgrowth of B16F10 and RMS brain tumors that were generated by direct intracranial implantation. Collectively, our results suggest that targeting CD38 in the melanoma TME provides a new therapeutic approach for melanoma treatment.

## INTRODUCTION

Melanoma is the deadliest skin cancer [1]. While early-detected melanoma is mostly curable by surgical excision, diffused metastatic disease is inevitably fatal [2-4]. The melanoma tumor mass is comprised of tumor cells and stroma, known as tumor microenvironment (TME). The interaction between tumor cells and TME modifies the microenvironment properties towards a phenotype that supports tumor progression and invasiveness [5, 6]. Moreover, the TME regulates the formation of metastasis [7].

The TME in cancers, including melanoma contains different types of cells including, endothelial cells, infiltrating immune cells and cancer-associated fibroblasts (CAFs) [5, 8, 9].

Tumor-cell directed drugs have been developed and shown to exert beneficial effects in various cancers [10]. However, due to the heterogeneity of tumor cells, even within a single tumor mass, this task is challenging. An alternative, yet complementary approach for cancer therapy is to target the TME cells.

Previously we showed that the ectoenzyme CD38 may serve as a useful microenvironmental target to inhibit glioma progression. Accordingly, we showed that targeting CD38 expression or its activity in the glioma microenvironment inhibited glioma progression and prolonged the lifespan of glioma-bearing mice [11-13]. CD38 is a nicotinamide adenine dinucleotide (NAD^+^) glycohydrolase and adenosine diphosphate (ADP)-ribosyl cyclase. It uses NAD^+^ to catalyze the formation of the calcium mobilizing metabolites: adenosine diphosphate ribose (ADPR), cyclic ADPR (cADPR) and nicotinic acid adenine dinucleotide phosphate (NAADP). Furthermore, due to its constitutively active NADase activity, CD38 is a major regulator of NAD^+^ homeostasis. CD38 can also act as a cell surface receptor [14]. CD38 regulates numerous processes e.g., cell activation, proliferation and migration of immune cells [14]. CD38 is also expressed in TME cells of extracranial tumors e.g., macrophages [15], T-cells [16] endothelial cells [17] and fibroblasts [18]. We therefore hypothesized that targeting CD38 in the microenvironment of extracranial tumors will inhibit their progression. To test this hypothesis we focused on melanoma, where TME is known to regulate tumor growth and metastasis [19-22]. Melanoma was induced in syngeneic mouse models comprised of C57BL/6J mice injected with B16F10 cells or Ret-mCherry-sorted (RMS) cells derived from a tumor in Ret-melanoma transgenic mice [23, 24] and transduced to express the fluorescent protein mCherry [25]. Our results show that targeting CD38 inhibited expansion of B16F10 or RMS primary tumors. The effect of CD38 targeting on B16F10 tumor progression involved increased cell death and reduction in the amount of CAFs and blood vessels, suggesting that loss of CD38 affects melanoma outgrowth through these effects. Moreover, loss of CD38 also inhibited occurrence of spontaneous pulmonary and brain metastasis as well as progression of brain tumors induced by intracranial injected B16F10 or RMS cells.

## RESULTS

### Loss of CD38 expression and activity attenuates B16F10 melanoma outgrowth

**Figure 1:**
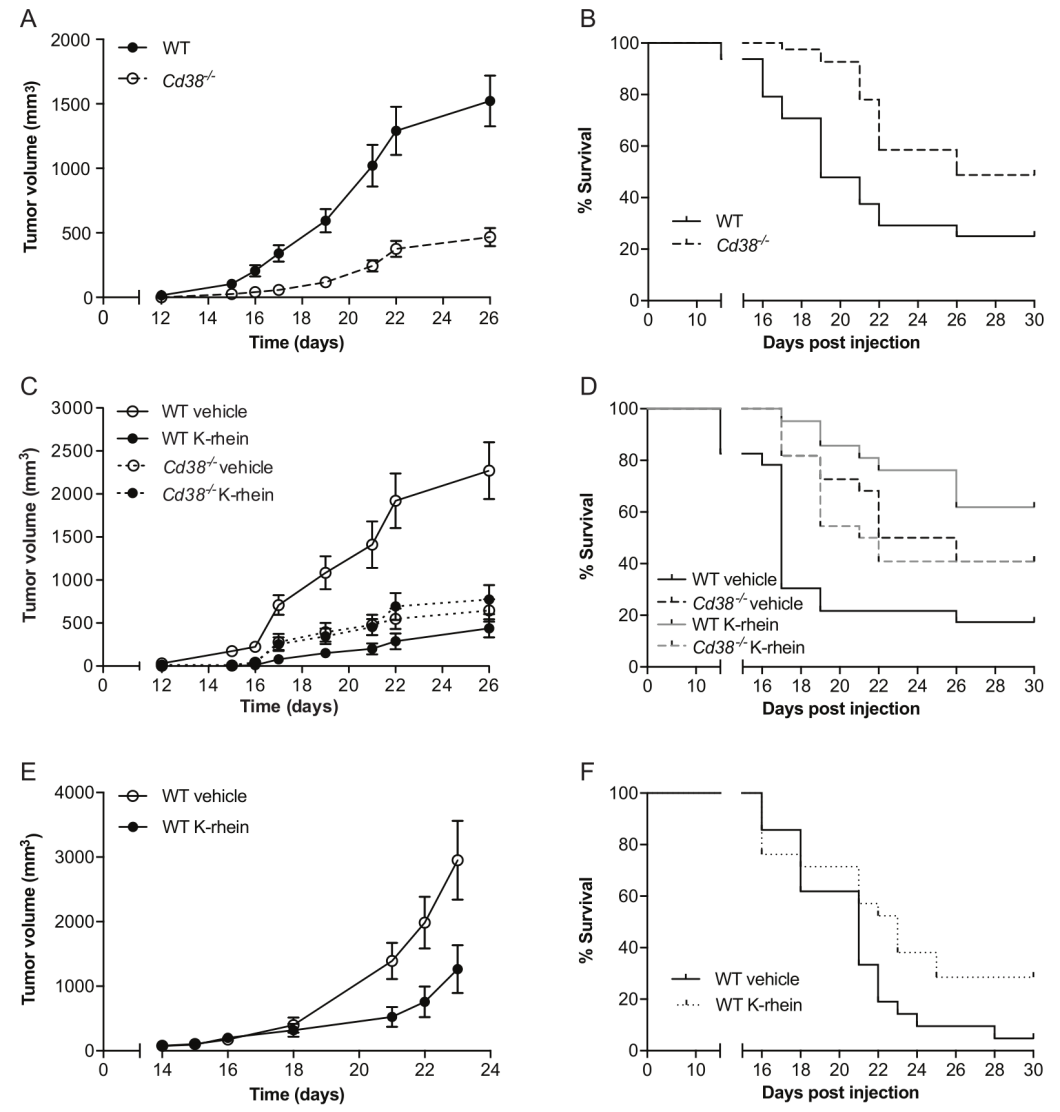
Targeting CD38 inhibits B16F10 melanoma progression WT and *Cd38*^*‒/‒*^ female mice SC-injected with B16F10 cells were untreated, treated with vehicle (H_2_O) or with K-rhein. Tumor volume was determined at the indicated time points. **(A)** Quantification of tumor volumes. **(B)** Kaplan-Meier survival curve. The results shown are from 4 independent experiments, values are presented as mean ± S.E.M (bars). Two-way ANOVA with repeated measures revealed a significant effect for *Cd38* genotype (*p* < 0.0001) (*n* = 48 WT, 41 *Cd38*^*‒/‒*^ mice). A log-rank test revealed a significant difference between the two groups (*p* = 0.0007). **(C, D)** Evaluation of the effect of K-rhein on tumor volumes (**C)** or survival (**D)** of B16F10 implanted WT and *Cd38*^*‒/‒*^ mice. Mice were pre-treated with K-rhein or vehicle one day before tumor cells implantation and then every 2-3 days. The results shown are from 3 independent experiments, values are presented as mean ± S.E.M (bars) Three-way ANOVA with repeated measures revealed a significant effect for time × treatment and for time × *Cd38* genotype × treatment (*p* < 0.0001) within subjects and for *Cd38* genotype × treatment between subjects (*p* < 0.0001). (*n* = 44 WT and 44 *Cd38*^*‒/‒*^ mice, of which *n* = 45 vehicle-treated and 43 K-rhein-treated mice). A log-rank test revealed a significant difference between vehicle and K-rhein treated WT (*p* = 0.0001) but not *Cd38*^*‒/‒*^ mice. **(E, F)** The effect of K-rhein administration after tumor establishment. WT mice were injected with B16F10 cells. After 14 days the mice were divided into two groups and treated with vehicle or K-rhein for additional 10 days. The results shown are from two experiments. (**E**) The effect on tumor volumes. Values are presented as mean ± S.E.M (bars). Two-way ANOVA analysis of tumor volume revealed a significant effect for time × treatment (*p* = 0.0001) (*n* = 18 vehicle- and 17 K-rhein-treated WT mice). Kaplan-Meier survival curve (**F**). A log-rank test revealed a significant difference between the two groups (*p* = 0.043).

To assess the role of CD38 in melanoma outgrowth, B16F10 melanoma cells were injected subcutaneously (SC) into WT or *Cd38*^*‒/‒*^ mice and tumor volume was measured at different time points following tumor cell implantation. The results show that tumor outgrowth was significantly reduced in the *Cd38*^*‒/‒*^ mice compared to WT mice and that at 26 days post-injection the average tumor volume in *Cd38*^*‒/‒*^ mice was significantly smaller than in WT mice (Figure 1A). Kaplan-Meier analysis (Figure 1B) revealed that loss of CD38 significantly prolonged survival of melanoma-bearing mice (median survival of *Cd38*^*‒/‒*^ mice was 26 days versus 19 days of WT mice). Next we examined if targeting CD38 enzyme activity recapitulates the effect of loss of CD38. WT and *Cd38*^*‒/‒*^ mice were injected with B16F10 cells and treated with vehicle or with the CD38 inhibitor K-rhein [12] and tumor volume was assessed at the indicated time points (Figure 1C). The results show that K-rhein significantly attenuated primary B16F10 tumor outgrowth in WT, but not in *Cd38*^*‒/‒*^ mice, indicating that the K-rhein inhibitory effect was CD38-dependent. Kaplan-Meier analysis revealed that K-rhein also significantly prolonged survival of melanoma-bearing WT, but not *Cd38*^*‒/‒*^ mice (Figure 1D). Next, we tested if treatment with K-rhein can inhibit growth of already existing melanoma tumors. Melanoma-bearing mice, 14 days after B16F10 cell injection, were divided into two groups harboring similar average tumor volume (∼100 mm^3^); one group was treated with K-rhein and the other with vehicle for the next 10 days. The results show that K-rhein significantly attenuated the subsequent tumor growth (Figure 1E) and prolonged survival (Figure 1F) of the melanoma-bearing mice (median survival of *Cd38*^*‒/‒*^ mice was 23 days versus 21 days of WT mice). Collectively, these results show that targeting CD38 by preventing its expression in the TME, or by inhibiting its enzymatic activity at the time of tumor cell implantation or after tumor establishment, attenuates B16F10 melanoma outgrowth.

### Pathological characterization of the B16F10 tumors in WT and *Cd38*^*−/−*^ mice

**Figure 2:**
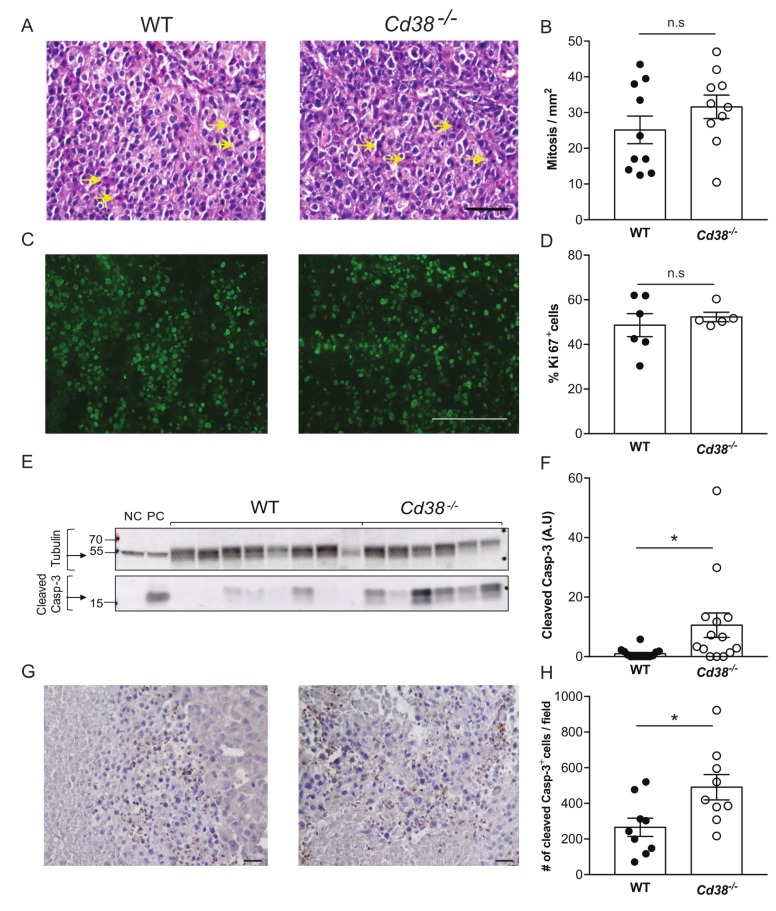
The effect of loss of CD38 on cell proliferation and cell death in late stage tumors (age matched) Paraffin and frozen sections or protein samples were prepared from WT and *Cd38*^*‒/‒*^ mice injected with B16F10 cells 28 days post injection. (**A***-***D**) Effect on proliferation. Proliferation was assessed using mitotic cell count in H&E stained sections (**A** and **B**) and Ki-67 staining (**C** and **D**). (**A**) Representative images of H&E stained tumors grown in WT and *Cd38*^*‒/‒*^ mice (scale bar = 100 μm); (arrows indicate representative mitotic cells). (**B**) Quantitation of the number of mitotic cells. The data presented are expressed as the mean ± SEM (bars) of the number of mitotic cells in mm^2^ (*n* = 10 mice from each group). n.s = not significant, Student’s *t* test. (**C**) Representative images of Ki-67 staining of tumors grown in WT and *Cd38*^*‒/‒*^ mice (scale bar = 200 μm) (**D**) Quantitation of the number of Ki-67 positive cells. The data presented are expressed as the mean ± SEM (bars) of the percentage of Ki-67-positive cells from total cells per field (*n* = 6 and 5 for WT and *Cd38*^*‒/‒*^ mice respectively). n.s = not significant, Student’s *t* test. Amount of cleaved (active) caspase-3 (Casp-3) in protein extracts prepared from the tumors (**E**, **F**). (**E)** A representative immunoblot probed for cleaved caspase-3 and β-tubulin. Negative control (NC) and positive control (PC) proteins were prepared from MEFs untreated or treated with staurosporine respectively. **(F**) Quantification of cleaved caspase-3 levels. Cleaved caspase-3 level is expressed as signal-intensity values (normalized to β-Tubulin). Expression levels were significantly higher in *Cd38*^*‒/‒*^ mice compared with WT (*p = 0.01; Student’s *t* test, n = 14 for each group). (**G)** Representative images of active caspase-3 staining in tumors originated from WT and *Cd38*^*‒/‒*^ mice (scale bar = 100 µm). (**H)** Quantification of cleaved caspase-3 levels. The data presented are expressed as the mean ± SEM (bars) of the average number of caspase-3 positive cells per field (*n* = 9 mice from each group). (*p = 0.02; Student’s *t* test).

Next, the effect of loss of CD38 on tumorigenic features was examined on B16F10 tumors, 28 days post-injection. First, we assessed cell proliferation and cell death. No significant difference in the number of mitotic cells (Figure 2A and 2B) or Ki-67 positive cells (Figure 2C and 2D) was observed in tumors grown in WT versus *Cd38*^*‒/‒*^ mice, indicating that the observed difference in tumor volume does not result from an effect on cell proliferation. However, the amount of active caspase-3 (an indicator of apoptotic cell death) in immunoblots and immunostained tumor sections of tumors grown in *Cd38*^*‒/‒*^ mice was significantly higher than in WT mice (Figure 2E and 2F), suggesting that loss of CD38 in the TME promoted cell death, which in turn reduced tumor size.

### The effect of loss of CD38 on blood vessels, necrotic area, peritumoral area and amount of CAFs

**Figure 3:**
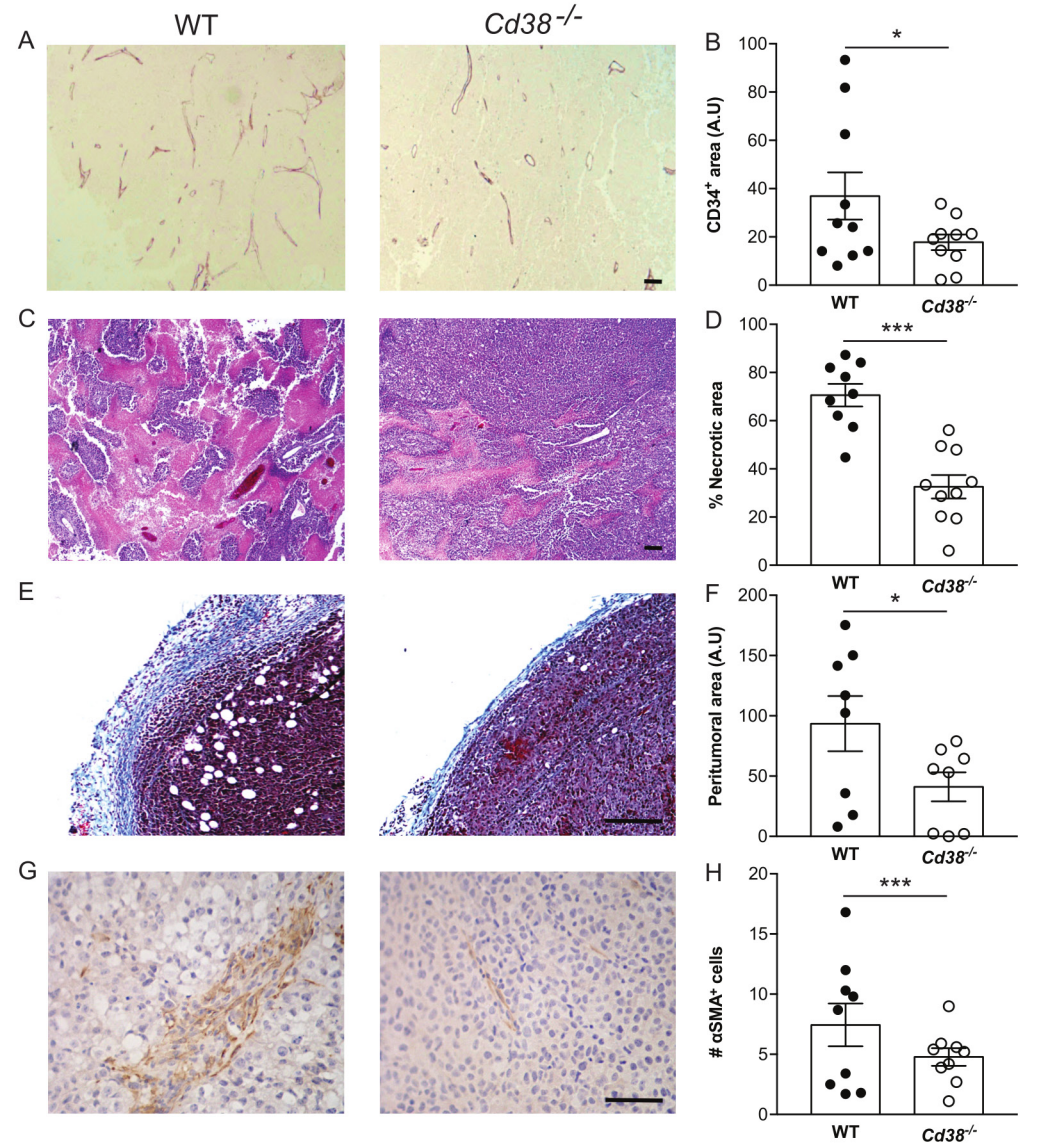
The effect of loss of CD38 on blood vessels, necrotic area, peritumoral area and amount of CAFs in late stage tumors **(A, B)** effect on blood vessels density. Paraffin sections were stained with anti-CD34 Ab. (**A)** Representative images of CD34 staining in tumors grown in WT and *Cd38*^*‒/‒*^ mice (scale bar = 500 µm). (**B)** Quantitation of the density of CD34 staining. The results are expressed as the average signal of CD34-positive area in each tumor section (*p = 0.03, Student’s *t* test*, n* = 10 mice from each group). (**C, D**) Effect on necrotic area. Necrotic regions were identified in H&E stained sections. (**C)** Representative images of necrotic area (pink) in tumors grown in WT and *Cd38*^*‒/‒*^ mice (scale bar = 500 μm). (**D)** Quantification of the percentage of necrotic area. The results are expressed as the average percentage of necrotic region in each tumor. Values are presented as mean ± S.E.M (bars) (***p 3.11 × 10^-5^; Student’s *t* test, *n* = 9 WT and 10 *Cd38*^*‒/‒*^ mice). (**E, F**) Effect on thickness of the peritumoral area. Collagen enriched peritumoral capsules were measured in Masson’s trichrome stained sections. (**E)** Representative images of tumors grown in WT and *Cd38*^*‒/‒*^ mice (scale bar = 250 µm). (**F)** Quantification of the peritumoral area. The results are expressed as the average area of peritumoral capsule in each tumor section (*p = 0.03; Student’s *t* test, *n* = 8 for each group). (**G, H**) Effect on the amount of CAFs. Sections were stained with anti-α-SMA Ab. (**G)** Representative images of α-SMA in the tumors grown in WT and *Cd38*^*‒/‒*^ mice (scale bar = 500 µm). (**H)** Quantitation of the density of α-SMA staining. The results are expressed as the average number of α-SMA in the counted regions. (**p = 0.001, Student’s *t* test, *n* = 9 mice from each group).

We further assessed the effect of CD38 on tumor vascularization. As shown (Figure 3A and 3B) the density of CD34 (endothelial marker) immunostaining in the tumors of WT mice was significantly higher than in *Cd38*^*‒/‒*^ mice. These results suggest that CD38 regulates tumor vascularization.

Assessment of H&E and Masson’s trichrome staining revealed that tumors grown in WT and *Cd38*^*‒/‒*^ mice contained necrotic and hypercellular regions (Figure 3C) and were encapsulated by collagen-containing peritumoral capsule (Figure 3D). However, the percentage of necrotic region (Figure 3E) and the average thickness of the peritumoral capsule were significantly higher (54, 60% respectively) in WT tumors compared to *Cd38*^*‒/‒*^ tumors (Figure 3F). The tumor capsule is a fibrotic tissue comprised mainly of collagen and fibroblasts. CAFs are known to play a supportive role in the progression of various solid tumors including melanoma, [26] we therefore assessed the level of CAFs (identified by the marker α-SMA) in tumors grown in WT and *Cd38*^*‒/‒*^ mice. The results show (Figure 3G and 3H) that the amount of α-SMA-positive cells was significantly lower in tumors grown in *Cd38*^*‒/‒*^ mice compared to WT mice.

### The effect of loss of CD38 on the properties of early stage and size-matched tumors

**Figure 4:**
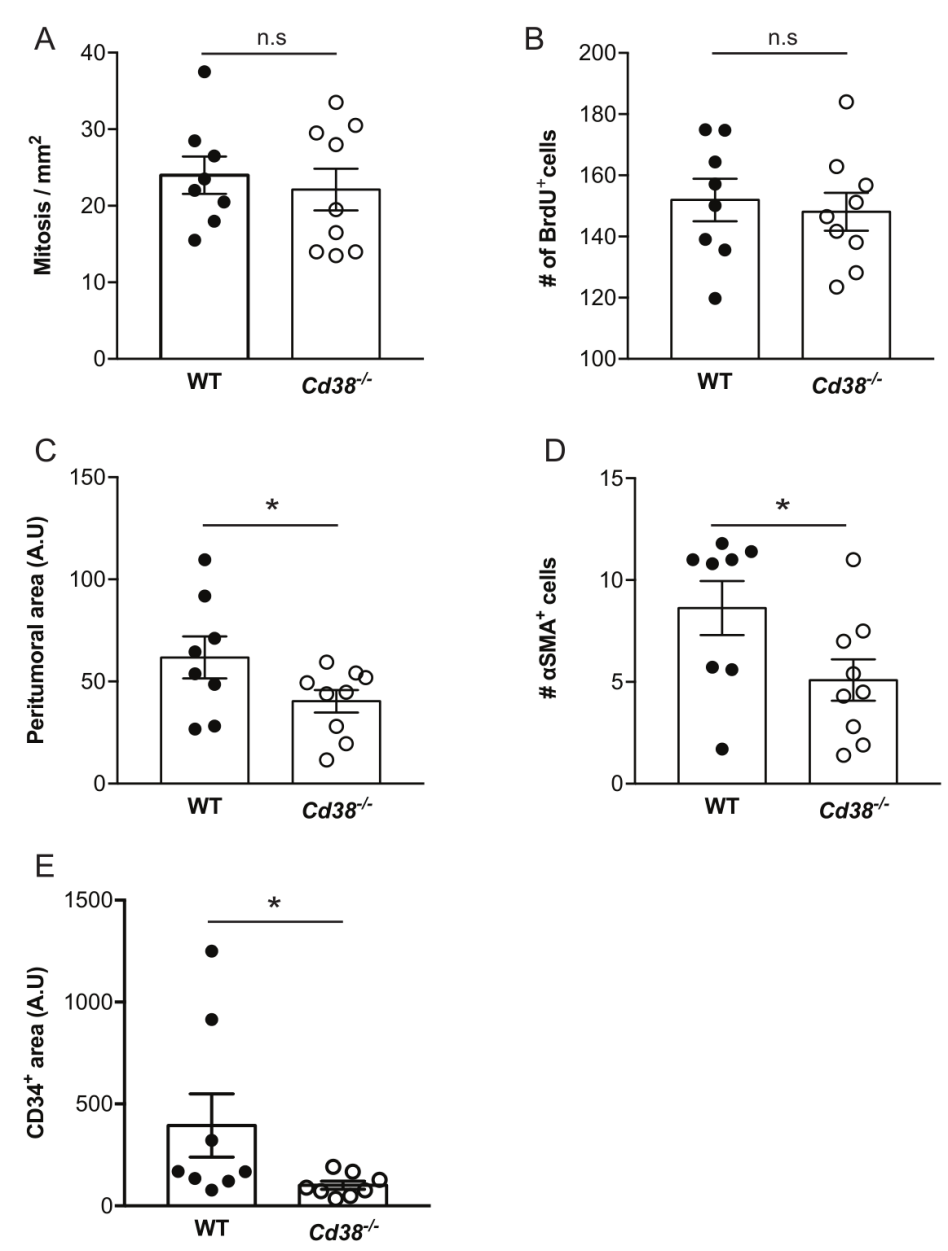
The effect of loss of CD38 on the properties of early stage and size-matched tumors Paraffin sections prepared from ∼140 mm^3^ tumors from WT and *Cd38*^*−/−*^ B16F10-injected mice were analyzed for the amount of mitotic cells, thickness of the peritumoral region, amount of CAFs and density of blood vessels. The representative images of these analyses are shown in Supplementary Figure 1. (**A)** Quantitation of the number of mitotic cells. The data presented are expressed as the mean ± SEM (bars) of the number of mitotic cells in mm^2^ (*n* = 8 WT and 9 *Cd38*^*‒/‒*^ mice). n.s = not significant, Student’s *t* test. (**B)** Quantification of the amount of BrdU staining. The data presented are expressed as the mean ± SEM (bars) of the average number of BrdU-positive cells per field. (*n* = 8 WT and 9 *Cd38*^*‒/‒*^ mice). n.s = not significant, Student’s *t* test. (**C)** Quantification of the peritumoral area. The results are expressed as the average area of peritumoral capsule in each tumor section (*p = 0.03; Student’s *t* test, *n* = 8 WT and 9 *Cd38*^*‒/‒*^ mice). (**D)** Quantitation of the density of α-SMA staining. The results are expressed as the average number of α-SMA in the counted regions. (*p = 0.02; Student’s *t* test*, n* = 8 WT and 9 *Cd38*^*‒/‒*^ mice). (**E)** Quantitation of the density of CD34 staining in the tumor area. The results are expressed as the percentage of CD34-positive area analyzed in each tumor section (*p = 0.04; Student’s *t* test*, n* = 8 mice from each group).

The tumors at 28 days post-injection are locally at advanced stage. To examine the effect of loss of CD38 at an earlier stage and to avoid possible size-dependent effects, we next assessed tumorigenic features in size-matched tumors (average volume 140 mm^3^) obtained at earlier time points after tumor cell implantation (average 14.8 and 18 days post-injection for WT and *Cd38*^*‒/‒*^ mice, respectively). Assessment of mitotic index, BrdU incorporation, thickness of the peritumoral capsule, amount of CAFs and density of blood vessels in tumor sections from the size-matched groups revealed that cell proliferation [assayed by mitotic index (Figure 4A and Supplementary Figure 1A) and BrdU incorporation (Figure 4B and Supplementary Figure 1B)] was similar in tumors grown in WT and *Cd38*^*‒/‒*^ mice, whereas thickness of the peritumoral capsule (Figure 4C and Supplementary Figure 1C), amount of CAFs (Figure 4D and Supplementary Figure 1D) and density of blood vessels (Figure 4E and Supplementary Figure 1E) were significantly lower in tumors grown in *Cd38*^*‒/‒*^ mice compared to WT mice. Thus, the inhibitory effect of loss of CD38 on thickness of the peritumoral capsule, amount of CAFs and density of blood vessels is already evident early in tumor outgrowth and this effect is tumor size-independent.

### Loss of CD38 attenuates primary RMS tumor outgrowth and spontaneous pulmonary and brain metastasis

**Figure 5:**
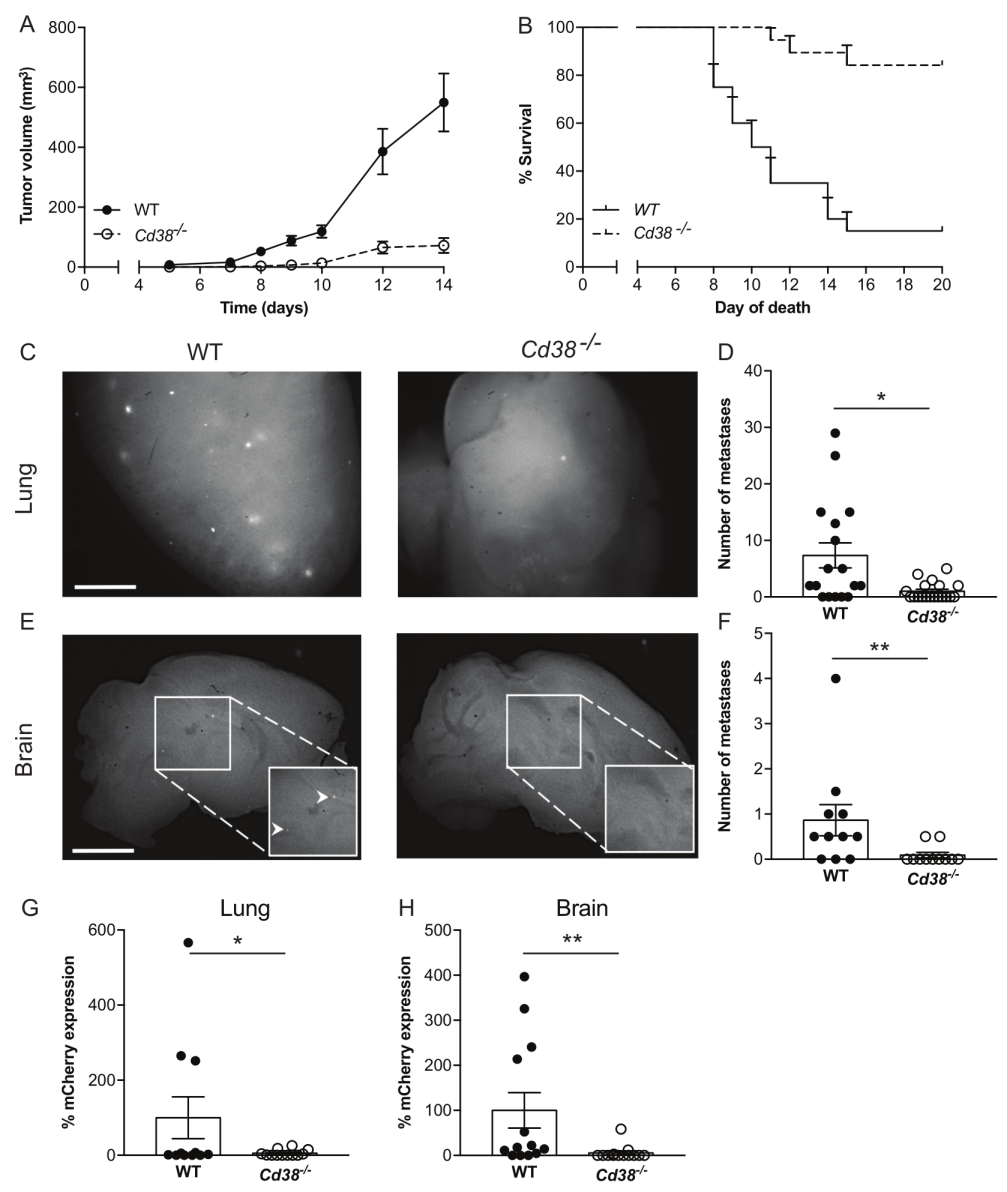
Loss of CD38 inhibits RMS melanoma primary tumor progression and spontaneous pulmonary and brain metastases (**A, B)** Effect of primary tumors. Male WT and *Cd38*^*‒/‒*^ mice were injected with RMS cells and tumor volume was determined at the indicated time points. (**A**) Quantification of tumor volume. (**B**) Kaplan-Meier survival curve. Results shown are from 2 independent experiments, values are presented as mean ± S.E.M (bars). Two-way ANOVA with repeated measures revealed significant effect for *Cd38* genotype (*p* < 0.0001) (*n* = 19 WT and 18 *Cd38*^*‒/‒*^). A log-rank test revealed a significant difference between the two groups (*p* < 0.0001). (**C-H)** Loss of CD38 inhibits spontaneous pulmonary and brain metastases. WT and *Cd38*^*‒/‒*^ mice were injected with RMS melanoma cells and processed for generation of spontaneous metastasis. Three months post-tumor excision, lungs and brains were harvested. Representative images of fluorescent mCherry foci in the lungs (**C**) and brains (**E**) of WT and *Cd38*^*‒/‒*^ (scale bar = 2 mm). Quantification of the number of metastatic foci in lungs (**D**) and brains (**F**). Results shown are from 2 independent experiments and are expressed as the number of foci per tissue. Values are presented as mean ± S.E.M (bars) [**p = 0.002 and *p = 0.02 for lungs and brains respectively; Student’s *t* test, *n* = 17 WT and 19 *Cd38*^*‒/‒*^ (lungs and brains)]. mCherry mRNA levels were determined in lungs (**G**) and brains (**H**) by qRT-PCR. The results are expressed as percentage of relative expression of mCherry normalized to *Hprt1*. Values are presented as mean ± S.E.M (bars) (*p = 0.04 and 0.01 for lungs and brain respectively; Student’s *t* test, *n* = 11 WT and 12 *Cd38*^*‒/‒*^ mice).

To test if the inhibitory effect of targeting CD38 on melanoma outgrowth is general, we utilized additional syngeneic mouse melanoma model, namely the RMS melanoma cells [25]. RMS cells were subdermally injected into WT or *Cd38*^*‒/‒*^ mice and primary tumor volume was measured at different time points. The results (Figure 5A) show that loss of CD38 substantially and significantly reduced RMS melanoma tumor volume (7.6-fold reduction in *Cd38*^*‒/‒*^ mice at 14 days post-injection) and prolonged survival of the RMS melanoma-bearing mice (Figure 5B). Thus, loss of CD38 inhibits melanoma outgrowth in two independent melanoma models.

Given the effect of loss of CD38 on primary melanoma, we next examined whether loss of CD38 may also inhibit occurrence of metastases. To this end, we utilized the RMS melanoma model, in which spontaneous lung and brain metastases occur [25]. WT and *Cd38*^*‒/‒*^ mice were subdermally injected with RMS cells, and primary tumors were surgically removed when they reached ∼1cm. Three months after removal of the primary tumor, metastatic load in the lungs and brain, two major sites of melanoma metastasis [25, 27], were assessed by determining: i) number of visible metastases (mCherry fluorescent foci). ii) mCherry mRNA level (indicative of the metastatic burden) by qRT-PCR. The results show that in *Cd38*^*‒/‒*^ mice, the number of mCherry fluorescent foci (Figure 5C and 5D for lung, Figure 5E and 5F for brain) as well as mCherry mRNA level in the lungs (Figure 5G) and brains (Figure 5H) were substantially and significantly lower than in WT mice. Thus, loss of CD38 also attenuates the occurrence (represented by number metastatic foci) and burden (represented by mCherry mRNA levels) of spontaneous RMS melanoma metastases.

### Loss of CD38 attenuates expansion of intracranially-injected RMS and B16F10 cells in the brain

**Figure 6:**
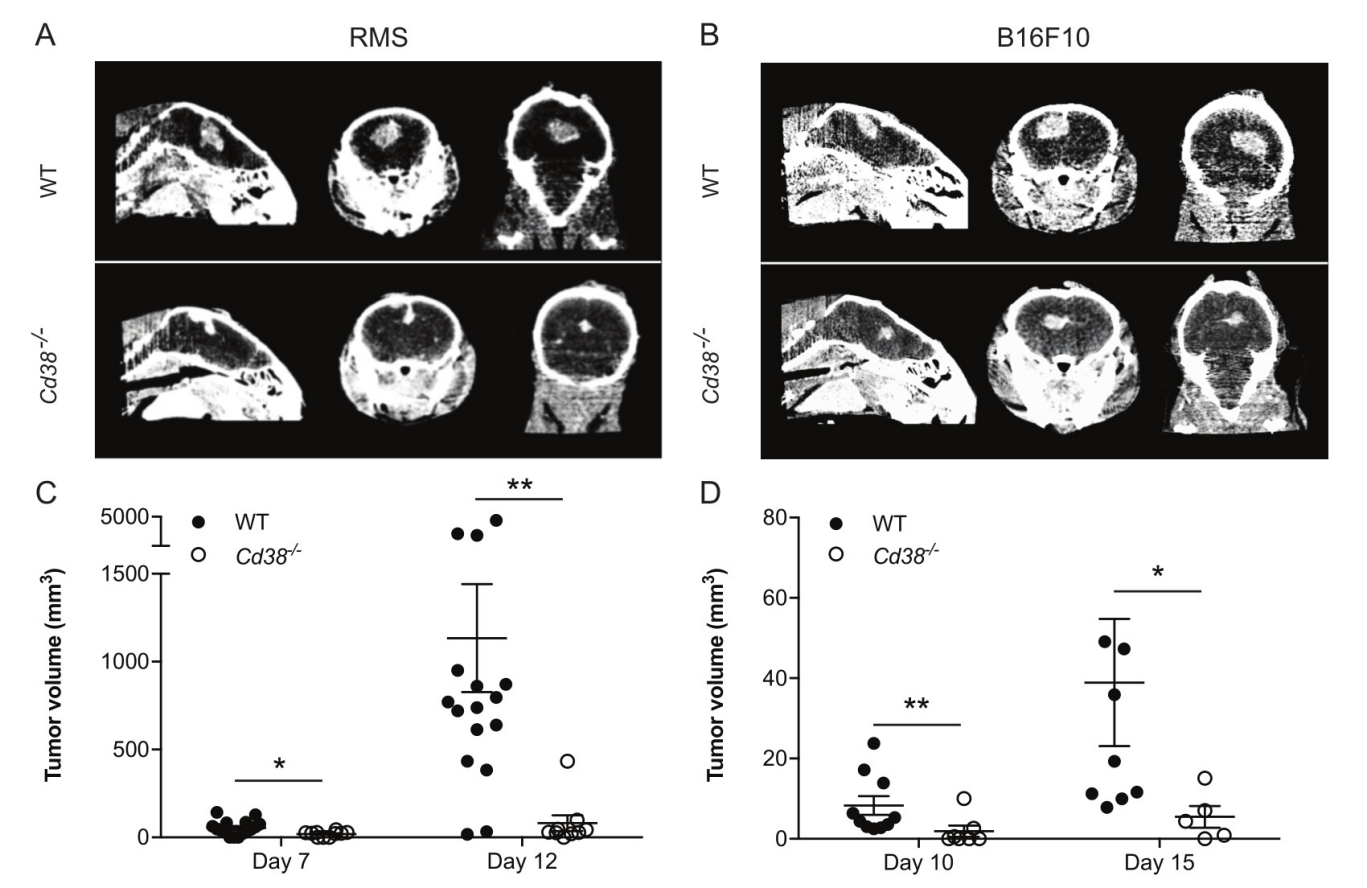
Loss of CD38 inhibits expansion of intracranially injected RMS or B16F10 cells WT and *Cd38*^*‒/‒*^ mice were intracranially injected with RMS or B16F10 cells. Tumor volume was measured by micro-CT at the indicated time points. Representative CT images of RMS (**A)** and B16F10 (**B)** tumors in WT and *Cd38*^*‒/‒*^ mice heads taken at 12 and 15 days respectively. Quantification of tumor volume in brains injected with RMS cells (**C)** or B16F10 cells (**D)** The results shown for RMS-injected mice are from 3 independent experiments, values are presented as mean ± S.E.M (bars). ANOVA with repeated measures revealed a significant effect for time × *Cd38* (*p* < 0.002) and for *Cd38* genotype for RMS-injected mice (*p* < 0.0001; *n* = 16 and 9 for WT and *Cd38*^*‒/‒*^ mice respectively). Bonferroni post-hoc test revealed a significant difference between WT and *Cd38*^*‒/‒*^ mice at day 7 (*p = 0.011) and day 12 (**p = 0.009). The results shown for B16F10-injected mice are from a representative experiment (out of three independent experiments which yielded similar results). Values are presented as mean S.E.M (bars) (Student’s *t* test revealed significant difference between the two B16F10 groups (**p = 0.006 and *p = 0.03, *n* = 10 and 7 for WT and *Cd38*^*‒/‒*^ mice respectively at day 10; Two mice in each group died after the first CT-procedure).

Next we asked whether the effect of loss of CD38 on occurrence of brain melanoma metastasis can be attributed to inhibition of expansion/colonization of tumor cells that have invaded the brain parenchyma. To this end RMS or B16F10 cells were intracranially injected into brains of WT or *Cd38*^*‒/‒*^ mice and the volume of the derived tumors was assessed by micro-CT at the indicated time points. The results show that the volume of the derived RMS (Figure 6A and 6C) or B16F10 (Figure 6B and 6D) tumors in *Cd38*^*‒/‒*^ brains was significantly smaller than in the WT brains. These results suggest that loss of CD38 inhibited expansion/colonization of melanoma cells in the brain.

## DISCUSSION

Targeting the TME is a promising therapeutic approach against various cancers. Using mouse melanoma models we demonstrate that targeting CD38 in the melanoma TME inhibited outgrowth of primary melanoma and reduced the occurrence of spontaneous pulmonary and brain metastases. Therefore, our results suggest that CD38 is a potential therapeutic target. This conclusion is based on the following findings: i) Loss of CD38 inhibited primary melanoma outgrowth in two different mouse models. ii) Treating B16F10 melanoma-bearing WT mice with a CD38 inhibitor restrained primary melanoma growth when applied together with tumor cells or after tumor occurrence. iii) In the RMS experimental model of spontaneous metastasis, the metastatic load was significantly lower in lungs and brains of *Cd38*^*‒/‒*^ mice. iv) Loss of CD38 inhibited the progression of brain tumors generated by intracranial injection of B16F10 or RMS melanoma cells.

How does loss of CD38 in the melanoma TME reduce tumor outgrowth? Loss of CD38 did not affect the amount of mitotic cells, but increased caspase-3 activation, suggesting that the reduction in tumor volume was mediated by increasing cell death and not by inhibiting cell proliferation. We speculate that the reduction in tumor volume is mainly due to death of tumor cells, however we cannot exclude the possibility that reduction in TME cells also contribute to this effect. Loss of CD38 also reduced the density of blood vessels and amount of CAFs. Since CAFs and blood vessels are known to facilitate tumor growth, it is possible that the inhibitory effect of loss of CD38 is mediated, at least partially, by reducing the amount of these tumor-supporting TME components. Notably, this effect was observed already at an early stage of tumor outgrowth and was size-independent, supporting the notion that these two TME components play a role in this effect. CD38 may also act via endothelial cells. It was suggested that CD38 regulates conversion of the PECAM1^+^/Sca1^+^/CD38^+^ endothelial cell population to α-SMA+ myofibroblast-like cells during skin wound repair [28]. Another study reported that apigenin and quercetin inhibited VCAM-1 expression in endothelial cells [29]. Since it has been shown that these flavonoids can act as CD38 inhibitors [30, 31], it is possible that they act on endothelial cells by inhibiting CD38 enzyme activity.

How does CD38 targeting affect the properties of TME cells? Since K-rhein inhibited B16F10 melanoma outgrowth in a CD38-dependent manner, it is feasible that the CD38 enzyme activity in the TME support melanoma growth. Inhibition of CD38 enzyme activity may cause: i) Reduction in intracellular Ca^2+^ levels, via inhibition of formation of cADPR, ADPR and NAADP. ii) An increase in NAD^+^ levels due to lack of CD38-mediated NAD^+^ hydrolysis. With respect to Ca^2+^, it was shown that the NAADP/Ca^2+^ pathway is critical for VEGF-induced angiogenesis in endothelial cells [32] and moreover that Ned-19, an NAADP pathway antagonist, inhibited B16 melanoma growth, vascularization and metastatization [33]. Although CD38 is not the only enzyme that produces NAADP [34] and the study of Favia et. al., [33] focused on the effect of NAADP on the tumor, but not the TME cells, a possible mechanism of loss of CD38 on melanoma could be a reduction in production of NAADP by the TME cells.

The effect of loss of CD38 can also be mediated by increasing NAD^+^ levels, which in turn may enhance the activity of NAD^+^ consuming enzymes e.g., PARP and sirtuins, that are known to be involved in cancer [35, 36] Additional pathway could be reduction in adenosine (ADO) levels, since the sequential action of CD38, CD203a (also known as PC-1), and 5′-ectonucleotidase CD73 produces adenosine.

We showed previously that targeting CD38 in the glioma microenvironment inhibited glioma progression, and that this effect was mediated by microglia/macrophages [11]. The novelty of the present study is the finding that targeting CD38 can also inhibit extracranial tumor (melanoma) outgrowth by a mechanism that involves reduction in the amount of CAFs and blood vessels. Thus, CD38 facilitates tumor growth by distinct microenvironment-dependent mechanisms in two different cancer microenvironments. Nevertheless, although our findings indicate that CD38 acts via different TME cells in different tissues and cancer types, it may still act via common pathway(s) e.g., increased NAD^+^ levels and/or reduced CD38 metabolites levels e.g., NAADP. In this regard it should be noted that the CD38 targeting effect can also be mediated by immune cells such as T cells. Accordingly it was shown that excess NAD^+^ can eliminate Treg cells by ATR2-mediated ADP-ribosylation of the P2X7 receptor, which in turn leads to death of the Treg cells [37, 38]. Since antitumor response is naturally suppressed by Treg cells, it is possible that CD38 targeting will promote anti-tumor response by eliminating Treg cells. In addition a recent study showed the CD38-NAD^+^ axis regulates anti-tumor T cell response in immunotherapy. Accordingly the anti-tumor activity of *ex vivo* generated Th1/17 cells was dependent on increased NAD^+^ levels, which was associated with decreased expression of CD38. Moreover, loss of CD38 from CD^4+^ T cells increased intrinsic NAD^+^ levels and led to metabolic reprograming of T cells with superior anti-tumor properties [39]. Lastly, CD38 targeting can affect T cells via reducing ADO production thereby inhibiting anti-tumor immune responses of T cells [40, 41]. In this respect it was shown that primary human melanoma cell lines suppress *in vitro* T cell proliferation through the CD38, CD203a and CD73 pathway [42].

We show here that loss of CD38 inhibited melanoma metastasis. This finding may have important clinical implications, as metastasis is the main cause of death from melanoma. The notion that CD38 targeting may serve as a potential therapeutic approach for patients with melanoma metastasis is supported by studies which showed that luteolin [43], apigenin and quercetin [29] (now known to be CD38 inhibitors [30, 31]) inhibited lung metastasis induced by intravenous injection of B16F10 cells to C57BL/6J mice. The effect of loss of CD38 on metastasis is unlikely to result from its effect on primary tumor growth, since the primary tumors were excised when reached similar size both in WT and *Cd38*^*‒/‒*^ mice. An alternative mechanism could be inhibition of progression/colonization of metastatic cells by the environment at the target organs. Indeed, at least in the brain, loss of CD38 inhibited the growth of intracranially injected melanoma cells.

Collectively, our results suggest that targeting CD38 in the melanoma TME may be a beneficial approach to treat melanoma both as a neoadjuvant treatment, to reduce tumor growth before resection of primary tumor, as well as for inhibiting the occurrence of metastasis.

## MATERIALS AND METHODS

### Reagents

Unless otherwise stated, reagents were purchased from Sigma-Aldrich (St. Louis, MO, USA). Rhein tri-potassium salt (K-rhein) was prepared as described [12]. Media were purchased from Invitrogen Life Technologies (Paisley, UK).

### Mice

C57BL/6J *Cd38*^*‒/‒*^ mice [44] were maintained at Tel Aviv University (TAU) animal facility. WT C57BL/6J mice were purchased from Envigo Jerusalem Israel (bl-6r252). All studies were performed according to protocols approved by the Animal Care and Use Committee of TAU.

### Cell lines

B16F10 melanoma cells stably express mCherry (herein B16F10), a kind gift from Prof. Ronit Satchi-Fainaro (TAU) were generated by infecting B16F10 cells with pQC-mCherry retrovirus. The cells were maintained in DMEM, supplemented with 10% fetal calf serum (FCS), 1% penicillin-streptomycin, 4 mM l-glutamine and 3 µg/ml puromycin at 37°C with 5% CO_2_. RMS cells [25] were grown in RPMI media supplemented with 10% FCS, 1% sodium pyruvate and 5% penicillin-streptomycin (all from Biological industries, Beit-Haemek, Israel) at 37°C and 5% CO_2_.

All cells were routinely tested for Mycoplasma using the EZ-PCR Mycoplasma Test Kit (Biological industries, Beit-Haemek, Israel).

### B16F10 cells injections and K-rhein treatment

B16F10 cells (50,000 / 100 µl) were S.C. injected into the right flank of WT or *Cd38*^*‒/‒*^ mice. 100 µl of K-rhein (10 mg/kg), or vehicle (H_2_O), was intraperitoneally injected either 24 h before tumor cells injection or when tumor reached ∼100 mm^3^ and then every 2-3 days. Tumor growth was monitored using caliper and ellipsoid volume was determined using the formula π/6 x (L x W x H).

### Preparation of B16F10 tumor samples

Two groups of tumors were analyzed: (i) advanced tumors (age-matched) and (ii) early stage tumors (size-matched). The first group was prepared from mice 28 days post B16F10 cells implantation and the second when the tumor volume reached ∼140 mm^3^. Melanoma-bearing WT or *Cd38*^*‒/‒*^ mice were sacrificed, perfused with PBS and tumors were excised and preserved either in 4% paraformaldehyde for sections’ preparation or snap-frozen in liquid nitrogen for protein extracts’ preparation.

### RMS melanoma model

Generation of primary tumor and spontaneous metastasis was performed as described [25]. Briefly 500,000 RMS cells were subdermally injected into the right flank of WT or *Cd38*^*‒/‒*^ C57BL/6J mice. Tumor volume was monitored using caliper. To model spontaneous metastasis, tumors were excised when one of the primary tumor dimensions reached 1 cm. Three months later, the mice were sacrificed, perfused with PBS and lungs and brains harvested.

### Analysis of metastasis foci

Fluorescence stereoscopic microscopy was used to count and quantify the number of mCherry foci in the lungs and brains.

### Determination of mCherry mRNA levels

Brains and lungs were homogenized and total RNA was prepared using EZ-RNA-II kit (Biological industries, Beit-Haemek, Israel). Quantitative RT-PCR (qPCR) was performed and values of mCherry mRNA were normalized to their corresponding *Hprt1* mRNA.

### Intracranial injection of B16F10 or RMS cells

10^3^ RMS or B16F10 cells were intracranially injected into brains of male or female (for RMS or B16F10 cells respectively) WT and *Cd38*^*‒/‒*^ mice, eight weeks of age as described [25].

### Computed tomography

Tumor volume of intracranial RMS- or B16F10-injected mice was determined by computed tomography (CT) imaging as described [12].

### Histology and immunostaining

Formalin-fixed, paraffin-embedded tumor blocks, cut into 6 μm thick sections were stained with H&E for evaluating necrotic areas and proliferation (mitotic cells) or with Masson’s trichrome to visualize collagen containing peritumoral capsule. Sections were also immunostained with anti-CD34 or anti-α-SMA mAb. Immunostaining was visualized with Rabbit-anti-Rat biotin-conjugated mAb for CD34 and BrdU, or Goat-anti-Rabbit biotin-conjugated mAb for α-SMA and caspase-3, and horseradish peroxidase (HRP)-conjugated streptavidin (Vectastain Elite ABC Kit, Vector Laboratories, Burlingame, CA, USA). For Ki-67 staining frozen sections were used. Tumors were submerged in Optimal Cutting Temperature Compound (O.C.T, Tissue-Tek Scigen Scientific, CA, USA) on dry ice and cut into 10 μm thick sections in a cryostat (CM1950, Leica). Sections were stained with anti-Ki-67 rabbit mAb. Immunostaining was visualized with fluorescently conjugated secondary goat-anti-rabbit, goat-anti-mouse AlexaFlour-488 (Thermo Fisher Scientific Waltham, MA, USA). Fluroshield Mounting Medium with DAPI (nuclei staining) (Abcam, Cambridge, MA, USA) was used for mounting.

### Image analysis

All analyses were done blindly using coded samples.

Analysis of necrotic regions- Non-overlapping images covering the entire tumor region were captured in H&E-stained sections. The necrotic area was expressed as percentage of necrotic region from the total measured tumor area.

Analysis of mitotic count- In each H&E section, the number of mitoses in 10 fields (X400 magnification) was analyzed covering 2 cm^2^ in each tumor by a certified pathologist.

Analysis of peritumoral capsule- Images of tumor sections stained with Masson’s trichrome (12-25 from each tumor) were captured at the tumor margins, and the average area of the capsule was calculated using ImageJ software package.

Analysis of CD34 and α-SMA staining- Images of 10 random fields of the tumor in a section were captured. The density of CD34 immunohistochemical staining was expressed as percentage of the area analyzed. The relative amount of α-SMA expressing cells was determined by manually counting α-SMA-positive cells in the fields examined. The results shown are expressed as the average number of α-SMA-positive cells per field.

Analysis of active caspase-3 and BrdU stain*ing*- Images of 10 random fields of the tumor in a section were captured with X40 magnification. The number of positive nuclei was counted using ‘find maxima’ function in ImageJ. The results shown are expressed as the average number of active caspase-3 or BrdU-positive cells per field.

Analysis of Ki-67 staining*-* Fluorescent images of Ki-67 and DAPI staining were captured in 10 random fields per section using X20 objective. The results shown are expressed as the percentage of Ki-67 positive cells from total cells per field [indicated by number of nuclei (DAPI staining)].

### BrdU Injection

When tumors reached the volume of ∼140 mm^3^, BrdU was i.p. injected (50 mg/kg) into WT or *Cd38*^*‒/‒*^ C57BL/6J. Two hours later, the mice were killed and their tumors were processed for immunostaining.

### Survival analysis

Following injection of the melanoma tumor cells, tumor volume and mouse weight were monitored. The survival endpoint of SC B16F10- or subdermal RMS cells-injected mice was defined when one of the tumor’s dimensions reached 1.5 cm. For intracranially RMS or B16F10 cells-injected mice, lack of physical activity and/or more than 15% reduction in body weight was defined as endpoint. The survival duration of the mice was analyzed by Kaplan-Meier survival analysis followed by a log-rank test.

### Immunoblotting

Protein extracts from 28 days post-injection B16F10 tumors were prepared. Proteins (50 μg/lane) were separated by 12% SDS−PAGE and electroblotted. Blotted membranes were cut at the 35 kDa molecular weight. The lower part was incubated with rabbit anti-cleaved caspase-3 mAb and the upper part with mouse anti-β-tubulin mAb followed by incubation with goat anti-mouse or goat anti-rabbit IgG peroxidase conjugate. The blots were developed and densitometric data were calculated using the ImageQuantTL program (GE Healthcare Life Sciences, Chicago, IL, USA). The caspase-3 signal was normalized to β-tubulin and quantified using EZQuant-Gel software.

### Statistical analysis

Analysis of variance (ANOVA) with repeated measures, 2-way ANOVA with repeated measures and 3-way ANOVA with repeated measures were used to compare tumor volume in WT and *Cd38*^*‒/‒*^ mice, untreated or treated with K-rhein. The survival duration of the mice was analyzed by Kaplan-Meier estimate followed by a log-rank test. Other experiments were analyzed by an unpaired Student’s *t* test; *P* < 0.05 was considered statistically significant.

## SUPPLEMENTARY MATERIALS FIGURE



## Abbreviations

(TME): tumor microenvironment
(RMS): Ret-mCherry-sorted
(CAFs): cancer-associated fibroblasts
(NAD^+^): nicotinamide adenine dinucleotide
(ADP): adenosine diphosphate
(ADPR): adenosine diphosphate ribose
(cADPR): cyclic ADPR
(NAADP): nicotinic acid adenine dinucleotide phosphate
(K-rhein): Rhein tri-potassium salt
(B16F10): mCherry B16F10
(FCS): fetal calf serum
(SC): Subcutaneous
(RT): reverse transcription
(qPCR): quantitative real time PCR
(CT): computed tomography
(H&E): Hematoxylin and Eosin
(ANOVA): analysis of variance
