# Supplementary material for: Stromal CD38 regulates outgrowth of primary melanoma and generation of spontaneous metastasis
Source: Oncotarget. 2018 Aug 7;9(61):31797–811. doi: 10.18632/oncotarget.25737 (PMC6112753; doi:10.18632/oncotarget.25737)
Supplement: Supplementary file 1 [file oncotarget-09-31797-s001.pdf]

Stromal CD38 regulates outgrowth of primary melanoma and generation of spontaneous metastasis

SUPPLEMENTARY MATERIALS

Analysis of metastasis foci

Brains and lungs were imaged using Zeiss Discovery.V12 SteREO fluorescence stereoscopic microscope (Carl Zeiss, Germany) with 543 nm filter for mCherry detection. Foci number was determined by counting number of foci in each brain microscopically. Images shown were captured using Plan S 1.0x FWD 81 mm objective.

Determination of mCherry mRNA levels

RNA extraction-Total RNA was purified using EZ-RNA-II kit (Biological Industries) according to the manufacturer's instructions. For brain RNA purification hemispheres were homogenized in 1 ml denaturation solution-A in M-tubes (Miltenyi Biotec) by gentle MACS-beads dissociator (Miltenyi Biotec). For lungs RNA purification, lungs were homogenized by TissueMaster 125 homogenizer (OMNI international).

Reverse transcription and quantitative real time PCR (RT-qPCR)-Reverse transcription was performed with qScript (Quanta Biosciences). qPCR analysis was performed using PerfeCTa SYBR Green FastMix, ROX (Quanta Biosciences) with the following primers: mCherry, forward 5'-GAA CGG CCA CGA GTT CGA GA-3', reverse 5'-CTT GGA GCC GTA CAT GAA CTG AGG-3'; *Hprt1*, forward 5'-GCG ATG ATG AAC CAG GTT ATG A-3', reverse 5'-ATC TCG AGC AAG TCT TTC AGT CCT-3'. The value of each mCherry transcript from each sample was normalized to the value of its corresponding *Hprt1* mRNA and the results were expressed as the relative expression of mCherry normalized to *Hprt1*.

Intracranial injection of B16F10 or RMS cells

Intracranial injection of tumor cells into WT and *Cd38*$^{-/-}$ mice was performed as previously described[1] with minor modifications. Briefly, eight weeks of age male or female WT and *Cd38*$^{-/-}$ mice, for RMS or mB16F10 cells respectively, were anesthetized and placed in a Kopf Stereotaxic Alignment System. An approximately 1 cm longitudinal cut was made in the scalp, and the skull was exposed. Then, 10^3 RMS or mB16F10 cells were injected 3 mm below the cortical surface through a hole drilled in the skull (1 mm posterior to the bregma and 1.5 mm lateral to it), at a rate of 1 µL/min. The scalp was sutured and the mice were allowed to recover in their cages.

Computed tomography

The tumor volume of intracranial RMS- or mB16F10-injected mice was determined at 7 and 12 or 10 and 15 days post tumor cells implantation respectively by computed tomography (CT) imaging. Briefly, mice were anesthetized by an i.p. injection of ketamine and xylazine and then 200 µL omnipaque (iohexol 350 mg/mL) were i.v. injected to the tail vein. The mice were then scanned by a TomoScope® Synergy micro-CT scanner, at a resolution of 100 µm with the following parameters: 40kV X-ray voltage, scan time: 90 s; 3 gantry rotations, radiation dose: 322 mGy/cm, each tube current: 1 mAmp. Tumor volume was determined using "3D Doctor" software version 4.0 (Able Software, Lexington, MA, USA), from the Windows 7 Platform software.

Image analysis

All the analyses were done blindly using coded samples.

Analysis of necrotic regions- Images of H&E-stained sections were captured using Nikon plan x 4/0.10 NA objective and a Nikon DS-5M camera (Nikon Instech, Tokyo, Japan). Non-overlapping images of the tumor were taken, covering the entire tumor section. The area of the necrotic regions was determined using Image-Pro Plus system (version 5.1; Media Cybernetics, Silver Spring, MD) by expressing the amount of the necrotic area as a percentage of the total measured tumor area.

Analysis of mitotic count- Two to four H&E-stained sections covering representative areas of the entire tumor were analyzed. In each section, the number of mitoses in 10 high power fields (X400 magnification) was analyzed covering 2 cm^2 in each tumor using Olympus BX41microscope.

Analysis of peritumoral capsule- Images of tumor sections stained with Masson's trichrome were captured at the tumor margins (12-25 images from each tumor, using Nikon plan 10X/0.25), and the average area of the capsule was calculated using ImageJ software package. When

images were taken at the edge of the section (and thus contained also white space), the analysis was performed only on the part which contained tissue.

Analysis of CD34 and α-SMA staining- Images of the tumor were captured using Nikon plan 10X/0.25 or 40X/0.65 objective (for CD34 or α-SMA respectively) and a Nikon DS-5M camera (Nikon Instech, Tokyo, Japan).

Immunofluorescence

Frozen tumor sections were incubated 10 min in room temperature with 4% PFA (Electron Microscopy Sciences), washed with PBS and incubated 10 min in 0.5% Triton X-100 (Sigma, St. Louis, MO, USA) followed by 1 h incubation at room temperature with 0.25% Triton X-100, 10% goat serum, 2% BSA (blocking solution). Then the sections were incubated overnight at 4° C with rabbit-anti-Ki-67 (Thermo Fisher Scientific Waltham, MA, USA) diluted 1:200 in blocking solution followed by addition of fluorescently conjugated secondary antibodies goat-anti-rabbit AlexaFlour-488 (A-11001, Thermo Scientific) diluted 1:700. After 1 h incubation at room temperature, the sections were washed with PBS and mounted with Fluroshield Mounting Medium with DAPI (Abcam, Cambridge, MA, USA #ab104140). The staining was visualized with EVOS FL fluorescent microscope at X20 magnification. Ten random fields of the tumor were captured. The total number of nuclei and Ki-67 positive nuclei was quantified with ImageJ.

Immunohistochemistry

Formalin-fixed, paraffin-embedded tumor blocks, cut into 6 μm thick sections were deparaffinized and boiled in antigen unmasking solution (citrate-based) (#H-3300, Vector Laboratories, Burlingame, CA, USA) for antigen retrieval. For intra-cellular antigens (aSMA, cas-3), sections were permeabilized with 0.25% Triton X-100 and washed with PBS. Then sections were blocked with 10% goat serum, 2% BSA (blocking solution) for 1.5 h in R.T, followed by overnight incubation at 4° C with one of the following primary antibodies: anti-CD34 mAb (1:200) (Cedarlane, Burlington, Canada) or anti-α-SMA (1:100) (Abcam, Cambridge, MA, USA), or anti-cleaved casapase-3 (1:400) (cell signaling, Danvers, MA, USA) or anti-BrdU (1:200) (ab6326, Abcam). Additionally, sections were treated with peroxidase block solution with 3% H_2O_2 for 15 min in R.T, and washed with PBS. Staining was visualized with Rabbit-anti-Rat biotin-conjugated mAb (1:200) for CD34 and BrdU, or Goat-anti-Rabbit biotin-conjugated mAb (1:200) for α-SMA and caspase-3, followed by horseradish peroxidase (HRP)-conjugated streptavidin (Vectastain Elite ABC Kit, Vector Laboratories, Burlingame, CA, USA), and developed with 3,3′-diaminobenzidine hydrochloride (DAB; Sigma, St. Louis, Mo, USA).

Immunoblotting

Tumors were excised from mB16F10 cells-injected mice, 28 days post-injection. The tissues were homogenized using Ystral homogenizer in lysis buffer containing 50 mM Tris-HCl (pH 7.6), 20 mM $MgCl_2$, 200 mM NaCl, 0.5% NP40, 1 mM DTT, and 1 mM antiproteases. For controls for active caspase-3 expression protein extracts were prepared from mouse embryonic fibroblasts (MEFs) untreated or treated with 100 ng/ml staurosporine for 16 h. 50 μg proteins from the different samples were separated by 12% SDS−PAGE and electroblotted onto supported nitrocellulose. Blotted membranes were cut into two parts at the 35 kDa molecular weight, blocked for 1 h in Tris-buffered saline/Tween-20 (10 mM Tris base, 150 mM NaCl, 0.05% Tween-20) containing 5% fat-free milk. Then the lower molecular weight part was incubated with the primary Rabbit anti-Cleaved Caspase-3 (Asp175) mAb (1:1000; Cell Signaling Technology #9664) and the upper molecular weight part with mouse anti-β-tubulin mAb (1:2500; Sigma #T4026) (to assess uniformly of the laded proteins loaded across the gel) overnight at 4°C. Washing of membranes three times (10 min each) with Tris-buffered saline/Tween-20 was followed by incubation for 1h at room temperature with the appropriate second Ab (goat anti-mouse or goat anti-rabbit IgG peroxidase conjugate; 1:10,000, Jackson ImmunoResearch Laboratories). The blots were developed using Luminata Crescendo Western HRP chemiluminescence Substrate (Millipore, MA, USA). Images were taken using the Amersham Imager 600 (GE Healthcare Life Sciences) and densitometric data was calculated using the ImageQuantTL program (GE Healthcare Life Sciences). The caspase-3 signal was normalized to β-tubulin and quantified by the use of EZQuant-Gel software.

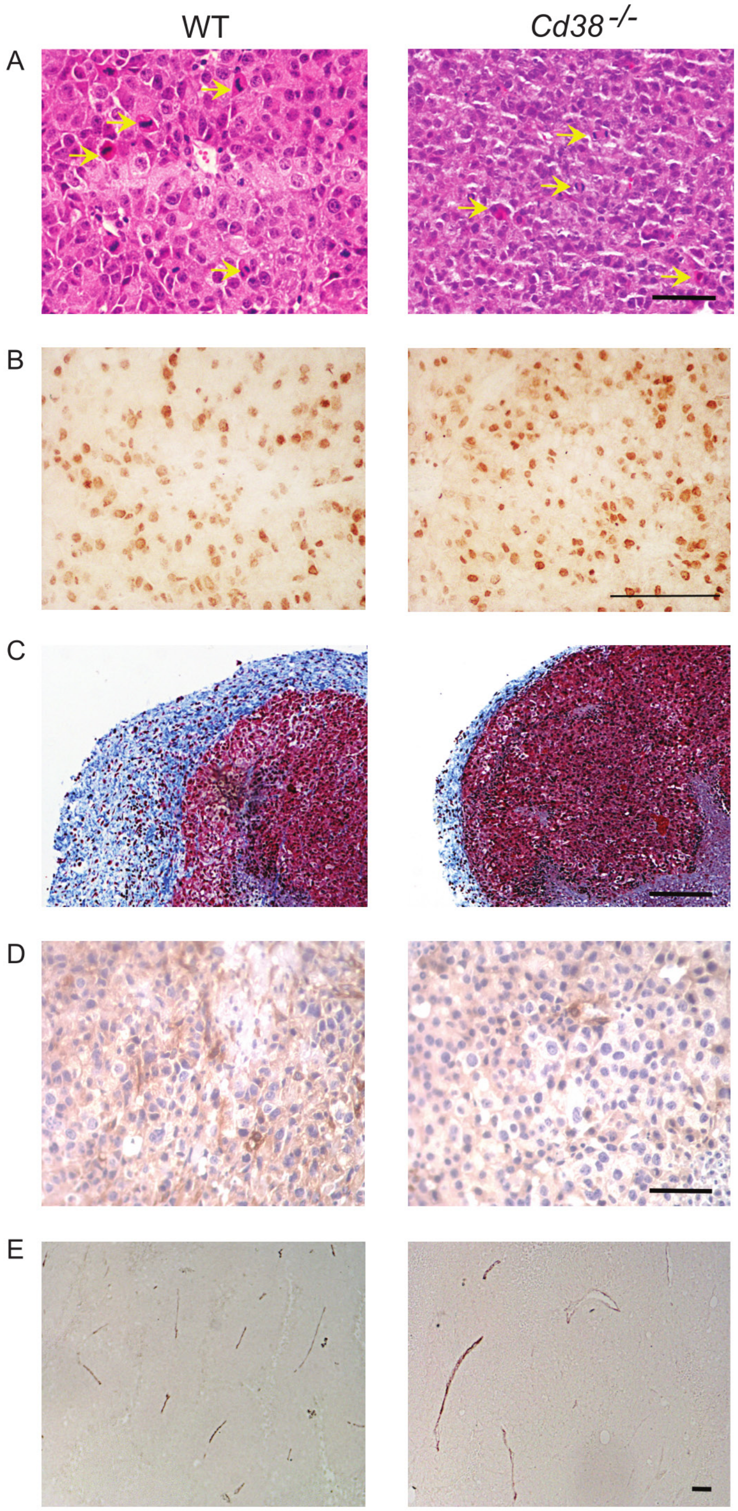

Supplementary Figure 1: The effect of loss of CD38 on the properties of early stage and size-matched tumors. WT and *Cd38*$^{-/-}$ mice were injected with mB16F10 cells. Tumors were removed when they reached the volume of ~140 mm^3 and then paraffin sections were prepared. Sections were processed for analysis of amount of mitotic cells, BrdU positive cells, thickness of the peritumoral region, amount of CAFs (α-SMA-positive cells) and density of blood vessels (CD34 staining) as described in Materials and Methods and in Figures 2 and 3. **A.** Representative images of H&E staining. Representative mitotic cells are indicated by arrows (scale bar = 100 μm). **B.** Representative images of BrdU staining (scale bar = 100 μm). **C.** Representative images of Masson trichrome staining (scale bar = 250 μm). **D.** Representative images of α-SMA staining in the tumors (scale bar = 500 μm). **E.** Representative images of CD34 staining in the tumor (scale bar = 500 μm).
